# When to cut your losses: Dispersal allocation in an asexual filamentous fungus in response to competition

**DOI:** 10.1002/ece3.5041

**Published:** 2019-03-12

**Authors:** Justin Y. Chan, Stephen P. Bonser, Jeff R. Powell, William K. Cornwell

**Affiliations:** ^1^ Evolution and Ecology Research Centre, School of Biological, Earth and Environmental Sciences University of New South Wales Sydney NSW Australia; ^2^ Hawkesbury Institute for the Environment Western Sydney University Penrith NSW Australia

**Keywords:** induced defence, informed dispersal theory, life history strategies, optimality, pycnidia, spores, trade‐offs

## Abstract

1. Fungal communities often form on ephemeral substrates and dispersal is critical for the persistence of fungi among the islands that form these metacommunities. Within each substrate, competition for space and resources is vital for the local persistence of fungi. The capacity to detect and respond by dispersal away from unfavorable conditions may confer higher fitness in fungi. Informed dispersal theory posits that organisms are predicted to detect information about their surroundings which may trigger a dispersal response. As such, we expect that fungi will increase allocation to dispersal in the presence of a strong competitor.
2. In a laboratory setting, we tested how competition with other filamentous fungi affected the development of conidial pycnidiomata (asexual fruiting bodies) in *Phacidium lacerum* over 10 days.
3. *Phacidium lacerum *was not observed to produce more asexual fruiting bodies or produce them earlier when experiencing interspecific competition with other filamentous fungi. However, we found that a trade‐off existed between growth rate and allocation to dispersal. We also observed a defensive response to specific interspecific competitors in the form of hyphal melanization of the colony which may have an impact on the growth rate and dispersal trade‐off.
4. Our results suggest that *P. lacerum *have the capacity to detect and respond to competitors by changing their allocation to dispersal and growth. However, allocation to defence may come at a cost to growth and dispersal. Thus, it is likely that optimal life history allocation in fungi constrained to ephemeral resources will depend on the competitive strength of neighbors surrounding them.

## 1 INTRODUCTION

Dispersal is a fundamental part of the life history of an organism. Effective dispersal can be an adaptive response to declining resource levels (Abrams, 2000; Holt, 2008) or increasing competition (Martorell & Martinez‐Lopez 2014). Dispersal can also be achieved through the production of reproductive propagules, a mode of dispersal vital to sessile organisms (Kinlan & Gaines, 2003). There are dispersal events seen in most species, and this phenomenon drives many spatial dynamics seen in the environment (Spiegel et al. 2010). Dispersal, patch dynamics, and species interactions together occur within communities and shape the colonization and extinction of species within a local patch (Hanski et al. 1998; Leibold et al., 2004; Lancaster & Downes, 2017). Thus, dispersal influences the ability of individual species to persist within a landscape (Johst, Brandl, & Eber, 2002). For most organisms, the ability to disperse is integral to persist in a fundamentally dynamic and often competitive environment (Edman, Gustafsson, Stenlid, Jonsson, & Ericson, 2004). However, the drivers that shape patterns in dispersal remain unclear for many groups of organisms, particularly for microorganisms such as fungi (Cadotte, Fortner, & Fukami, 2006; Fuhrman, 2009; Lancaster & Downes, 2017; Nemergut et al., 2013).

Informed dispersal theory posits that organisms integrate information about their internal condition and external environment, with the dispersal option weighed against the costs of remaining in the current environment and triggered when costs reach a certain level (Clobert, Galliard, Cote, Meylan, & Massot, 2009). The increase in allocation to dispersal in plants can be influenced by environmental stress (Martorell & Martinez‐Lopez 2014) and competitive conditions (French, Robinson, Smith, & Watts, 2017; Tabassum & Bonser, 2017), where theses adversities induce higher allocation to reproduction. However, these results may not be directly applicable to understanding the drivers of dispersal in microbial systems. For instance, in many situations, key resources in plants do not deplete over the course of an individual's lifespan (e.g., light) or are accessed by organs exploring new patches (e.g., nutrients). In contrast, for many microbial systems, the fundamental resources for growth (such as carbon, nitrogen, phosphorus) deplete at the scale of the spatial extent and lifespan of an organism. It is not clear the extent to which predictions from plant systems may be extrapolated to much smaller, heterotrophic organisms like fungi.

Competition for resources is common in saprotrophic fungi (Boddy, 2000). Fungi primarily compete over space, and the control of resources within territories (Hiscox, Savoury, Vaughan, Müller, & Boddy, 2015). Access to resources held by other fungi often requires combative antagonism to breach an already occupied space. Variation in competitive ability is common, with distinct hierarchies formed by interactions bound in a substrate (Boddy, 2000; Stenlid & Gustaffson 2001; Haňáčková et al., 2015, Van der Wal, Klein Gunnewiek, Cornelissen, Crowther, & Boer, 2016). Thus, delaying dispersal through delaying reproduction may result in low fitness if a fungal colony cannot withstand the combative assault of a competitively superior fungus. This presents a scenario where the benefits of remaining stationary diminish over time as resources become depleted, and the costs of defending a territory overtake the costs of dispersal (Bonte et al., 2012; Clobert et al., 2009). Increased allocation to dispersal in response to competition has been demonstrated in plants (Bonser et al. 2013; Fazlioglu, Al‐Namazi, & Bonser, 2016), and this response is associated with a strategy of escaping intense competition, but whether fungi increase allocation to dispersal in response to competition is still unknown.

Fungi are similar to plants in that they are a modular organism with the capacity to respond to stimuli at the growing point of each hyphal tube (Lee, Fricker, & Porter, 2016). While little is known about the shift to dispersal, fungi have the ability to dynamically allocate resources and translocate nutrients through their mycelia (Philpott, Prescott, Chapman, & Grayston, 2014; Tlalka, Bebber, Darrah, Watkinson, & Fricker, 2008). Fungi tend to be highly responsive to environmental factors, altering their colony growth form in response to resource pools (Heaton et al., 2012), fungivore attack (Crowther, Jones, & Boddy, 2011), and interspecific mycelial interaction (Rotheray, Jones, Fricker, & Boddy, 2008). We tested if fungi are able to alter allocation of resources to dispersal as a response to a decrease in environment quality or increasing competition as a strategy to persist as a sessile organism in an unstable environment.

Here, we take advantage of a system for studying allocation to air‐borne dispersal using an asexual filamentous fungus, *Phacidium lacerum*. *Phacidium lacerum*, an ascomycetous pathogen and filamentous fungus of the order Helotiales, is known to infect pine trees (Nawrot‐Chorabik, Grad, & Kowalski, 2016) and Rosaceae fruits (Wiseman, Kim, Dugan, Rogers, & Xiao, 2016) with no previously documented presence in decaying wood. The primary form of dispersal is through the production of ascomata or pycnidial conidiomata (Supporting information Figure S1) emerging from the surface of infected plant material, and the subsequent release of ascospores or asexual conidia (Crous, Quaedvlieg, Hansen, Hawksworth, & Groenewald, 2014). It was chosen as a suitable species as it is capable of developing pycnidia in vitro in a Petri dish on growth media. This property allows us to quantify the density of pycnidia over colony area as a proxy of allocation to air‐borne dispersal, as such, it is the focal species of this study. With this new model system for fungal allocation to air‐borne dispersal, we can quantify allocation to dispersal with and without a competitor present and begin to connect dispersal to an emerging understanding of fungal life histories. We predict that (a) competition will induce early reproduction in a fungal colony, and (b) competition will induce high allocation to reproduction in a fungal colony.

## 2 MATERIALS AND METHODS

### 2.1 Study species

We extracted isolates of *Phacidium lacerum* (Isolate Face008) from rotting *Eucalyptus tereticornis* logs in Richmond, NSW (33°37′04.0″S 150°44′25.3″E) in February 2016. The site is in remnant Cumberland Plain woodland dominated by *Eucalyptus tereticornis*. The isolates were collected by splitting the wood with a sterilized chisel and extracting wood chips from the center of the logs. We placed the wood chips on 2% malt extract agar (MEA) and subcultured from the emerging hyphae until we attained a pure culture. We extracted DNA from the growing hyphae from fungal cultures using DNeasy Plant Mini Kit (Qiagen, Chadstone, Victoria, Australia) as per the manufacturer's instruction. We amplified the ITS (ITS1F & ITS4) region of rDNA (Thompson, Thorn, & Smith, 2012) through PCR amplification and analyzed the amplicons using a ABI3500 Genetic Analyser (Applied Biosystems, Life Technologies, Mulgrave, Victoria, Australia). The species identity was confirmed by conducting a BLAST search against the NCBI Nucleotide database.

### 2.2 Isolating competitors

To test whether *P. lacerum* responds to competitive interactions, we selected three basidiomycete fungi capable of degrading wood. A* Phanerochaete *sp. (Face061) was extracted from the same wood blocks as *P. lacerum*. We obtained two more fungal isolates from existing collections in the Hawkesbury Institute of the Environment (Western Sydney University, Richmond), *Omphalotus *sp. (MT5A) and an unidentified cord‐forming basidiomycete (HWK05). We selected these species as they are likely able to impose competitive pressures on *P. lacerum* over the course of the experiment as they tested positive with a lignin‐guaiacol test, indicating that they were able to produce oxidative enzymes (Hiscox & Boddy, 2017). All fungal isolates were maintained on 2% MEA at 4°C (see Figure 4 for colony morphology).

### 2.3 Experimental design

To test whether a fungus alters dispersal allocation as a result of competitive interactions, we set up multiple pairwise interspecific and intraspecific interactions with the focal species *P. lacerum *on Petri dishes and growing on 2% MEA. Our experiment consisted of five treatments with *P. lacerum*: (a) *P. lacerum* alone, (b) *P. lacerum *in intraspecific competition with a genetic clone of itself, (c) the focal species in interspecific competition with the *Phanerochaete *sp. isolate, (d) *Omphalotus *sp. isolate, or (e) with the isolate HWK5. We had eight replicates of each treatment with a total of 40 treatment plates.

Prior to the experiment, all species were inoculated onto H_2_O agar (10 mg/L) to normalize hyphal density between species. The cultures were allowed to establish on the H_2_O agar for 7 days before 5 mm diameter plugs were taken from the growing edge of the colonies and inoculated onto Petri dishes (9 cm diameter) with 2% MEA. Focal species in the alone treatment were inoculated onto the center of the petri dish. For each of the competition treatments, *P. lacerum* was inoculated 2.25 cm away from the edge of the Petri dish, while the competitor was inoculated 2.25 cm from the center, opposite to the *P. lacerum *inoculation such that both inocula had equal area to develop (see Figure 4). All Petri dishes were incubated in the dark at 25°C.

### 2.4 Image analysis

We assessed the Petri dishes every day for colony growth and measured colony growth rate every day by tracking colony size. We measured colony growth rate by measuring the log difference in colony area every 24 hr to calculate relative growth rate (Lambers & Poorter, 1992). After the first emergence of pycnidia, two days following inoculation, we conducted a census of the number and location of pycnidia every 12 hr by marking the position of pycnidia on the back of the Petri dish with an acetate sheet and black permanent marker. For each experimental plate, we measured the number of pycnidia, and colony radius to estimate reproductive allocation as number of pycnidia relative to colony size. This allowed us to obtain a density measure to account for differences in colony size as a result of interaction. An image of this sheet was captured after every time point using a Canoscan LiDE 210 scanner (300 dpi resolution) to track accumulation of pycnidia over the course of the experiment. We monitored pycnidial formation over 8 days (180 hr), which was the time taken for pycnidial development to reach the edge of the colony when the focal species grown alone. This way, we could compare both the timing and the extent of dispersal allocation across the experimental treatments against a colony growing alone. The experiment ran for a total of 10 days.

We processed the scans of the pycnidia after every sampling period using ImageJ (NIH, USA). We manually selected for every black dot using color thresholding in the RGB color space, thus creating a binary image. We then used the particle analysis package in ImageJ to automate counts of pycnidia from the binary image. We specified a minimum size of 20‐pixel units to discount any spurious marks.

### 2.5 Statistical analysis

To test the hypothesis of a shift in the timing of allocation to dispersal in response to competition, we fit a survival analysis model (also called a time‐to‐event model) to data for when *P. lacerum* produced at least 100 pycnidia. This model estimated the time to at least 100 pycnidia, along with a treatment effect for this variable and a significance test for the treatment effect. Analysis was carried out in the SURVIVAL package (Therneau, 2015) in R (v.3.4.2). To test whether *P. lacerum* produce more pycnidia as a response to competition, we ran a one‐way analysis of variance (ANOVA) with treatment as the competition block in R (v.3.4.2) for final pycnidial density at hour 180. Tukey's HSD post hoc tests were used to assess significant differences between treatments. To estimate the slopes of the trade‐off between growth and dispersal for the different treatments, we fit a longitudinal two‐level mixed model in which we have repeated measures of the same petri dish (see Diggle, Heagerty, Liang, & Zeger, 2002)**.** The goal of the model was to test for differences in the relationship between dispersal allocation and RGR we tested this model both with and without first order temporal correlation structure, and results were very similar. Results are reported for the simpler model without correlation structure. RGR values were transformed to z‐scores to facilitate model convergence. The number of pycnidia was the response variable and model fitting was done with a Poisson error term via penalized quasi‐likelihood in R (v.3.4.2) within the MASS package (Venables & Ripley, 2002). Reference level for the model was the control treatment and post hoc significance testing for differences in intercepts and slopes were done via summary.glmmPQL function in the MASS library (Venables & Ripley, 2002).

## 3 RESULTS

### 3.1 Colony growth and contact with competitors

**Figure 1:**
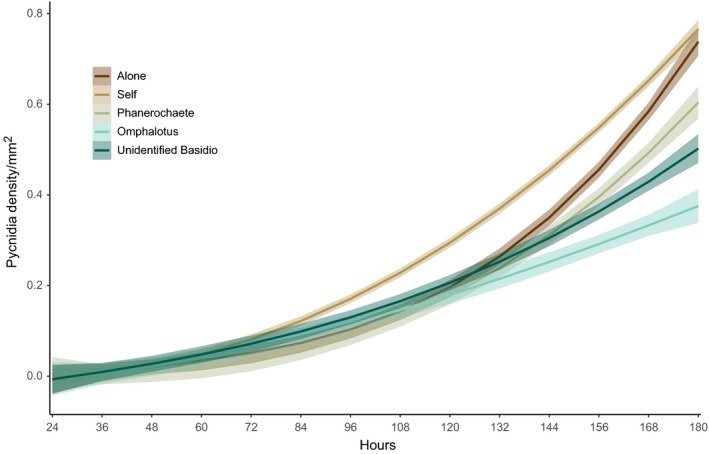
Spline ± *SE* of pycnidia density in mm^2^ over 7 days. *P. lacerum* came into contact with other colonies of *P. lacerum* at 48 hr. *Omphalotus* sp. and Isolate HWK5 came into contact with *P. lacerum* at 72 hr. *Phanerchaete *sp. came into contact with *P. lacerum* at 96 hr. *P. lacerum* growing alone came into contact with the Petri dish edge at 120 hr

The hyphal front of the colonies of *P. lacerum *came into first contact earliest in the intraspecific competition treatment at two days (Figure 1). This was followed by the focal species in interspecific treatments at three (*Omphalotus *sp. and Isolate HWK5) and four days (*Phanerochaete *sp.) for colony contact. When *P. lacerum *was grown alone, it took five days on average for the hyphal front of the colony to reach the edge of the Petri dish from the inoculum in the center of the Petri dish.

We observed different competitive outcomes with the focal species and its competitors. By the end of the experiment, the *P. lacerum *colonies grew larger than *Phanerochaete *sp. colonies. The hyphal front of the colonies of *P. lacerum* did not breach into the space held by the neighboring *P. lacerum *colony during intraspecific competition. We did not observe colonies of *P. lacerum *breaching into the space of the neighboring colony during interspecific competition with Isolate HWK5. *Phacidium lacerum *was reduced in size when in competition with *Omphalotus *sp., with *Omphalotus *sp. growing and extending into the space held by the colony of *P. lacerum *(Figure 4). *Phacidium lacerum* responded to colony contact with Isolate HWK5 and *Omphalotus *sp. with a conspicuous pigmentation of the leading hyphal front at the point of contact (Figure 4)**.**

### 3.2 Allocation to dispersal as a response to competition

**Figure 2:**
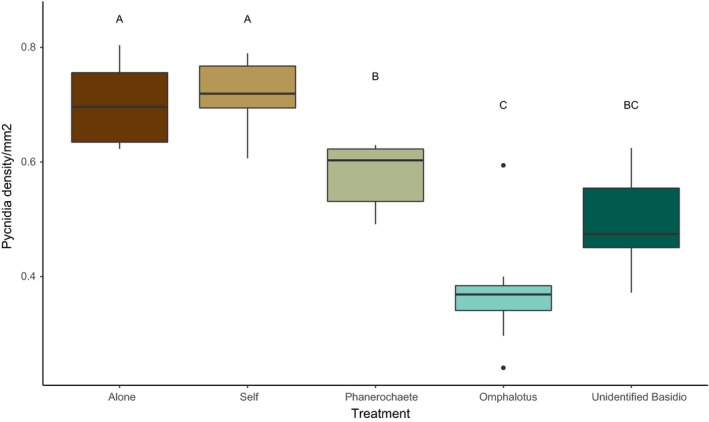
Pycnidial density per mm^2^ among treatments at 180 hr. Significant differences in pycnidial density denoted by letters determined by Tukey's *post hoc* tests. Pycnidial density in *P. lacerum* colonies is significantly lower when in interspecific competition than *P. lacerum *colonies in intraspecific competition or in the absence of interaction

On average, pycnidia appeared at 67 (±1.05 *SE*) hours across all treatments (Figure 1), with no significant difference in timing of emergence of pycnidia as a response to competitor presence or identity (survival analysis, *p* > 0.05 for treatment). Pycnidial density varied among treatments at the end of the experiment (180 hr). Overall, we found a significant difference in pycnidial density between treatments (Figure 2, ANOVA, *F*_4,42_ = 40.95, *p* < 0.001). Post hoc Tukey's HSD tests showed that *P. lacerum* had significantly reduced pycnidial density when experiencing interspecific competition with the unidentified basidiomycete (Tukey's HSD, *p* < 0.001), *Phanerochaete *sp. (Tukey's HSD, *p* < 0.01) and *Omphalotus *sp. (Tukey's HSD, *p* < 0.001). We did not observe a reduced allocation to dispersal when *P. lacerum* was in intraspecific competition with a genetic clone of itself, with no significant difference in the density of pycnidia in the colony (Tukey's HSD, *p* = 0.96).

### 3.3 Trade‐offs in allocation

**Figure 3:**
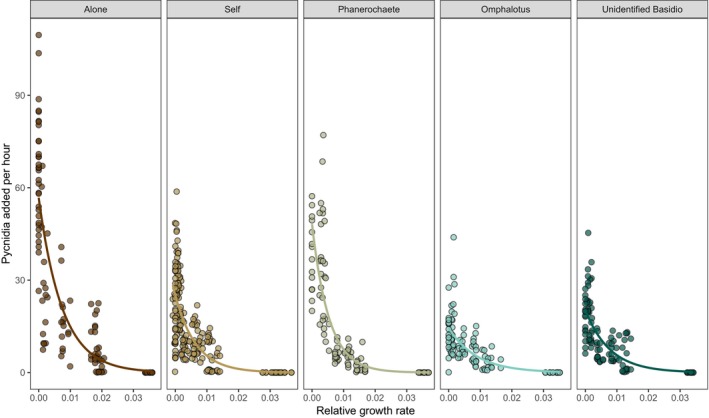
Comparison of relationship between pycnidia development per 12 hr for *P. lacerum* against relative growth rate. Note that time runs opposite to relative growth rate (moving left). There were no significant differences in the slope of this relationship between when *P. lacerum *was grown alone versus when *P. lacerum *was in intraspecific competition, or in competition with *Omphalotus *sp., or in competition with Isolate HWK5. The slope was significantly steeper when *P. lacerum* was in competition with *Phanerochaete *sp.

We observed a negative relationship between growth rate and dispersal allocation for all treatments (Table 1). We found that there was no significant difference in the slope of this relationship between when *P. lacerum* was grown alone versus when *P. lacerum *was experiencing either intraspecific competition (*p* = 0.852, GLM), and interspecific competition with *Omphalotus *sp. (*p* = 0.058, GLM) or interspecific competition with Isolate HWK5 (*p* = 0.308, GLM). However, the slope of the relationship was significantly steeper when in competition with *Phanerochaete *sp. (Figure 3, *p* < 0.0001, GLM).

**Table 1:** Longitudinal two‐level mixed model with repeated measures of individual Petri dishes to test for differences in the relationship between dispersal allocation and RGR

	Coefficient	*SE*	*p*	*df*
Main effects				
Alone (Reference)	3.694	0.228	<0.0001	393
Self	−0.33	0.116	0.007	42
Phanerochaete	−0.359	0.121	0.005	42
Omphalotus	−0.721	0.13	<0.0001	42
HWK 5	−0.512	0.124	0.0002	42
Interactions				
Alone (Reference)	−0.151	0.063	0.018	393
Self	−0.019	0.105	0.852	393
Phanerochaete	−0.559	0.132	<0.0001	393
Omphalotus	0.234	0.123	0.058	393
HWK 5	0.119	0.116	0.308	393

Note

Reference level for the model was the control treatment with the focal species alone.

## 4 DISCUSSION

Allocation to reproductive dispersal was not observed to increase under competition. Daily censuses also did not detect an earlier shift to allocation in response to interspecific competition. This is contrary to how annual plants respond to competition (Martorell & Martinez‐Lopez et al. 2014; Fazlioglu et al., 2016). The production of pycnidia began across treatments prior to colony contact, with no significant difference in production even when *P. lacerum* was grown alone (Figure 1). This suggests that the timing of allocation to dispersal begins prior to interaction and that allocation to dispersal occurred even in the absence of competition (Figure 1). We also did not find support for our predictions that fungi will allocate relatively more to reproductive dispersal in response to competition. In contrast, we found support for the opposite effect, where interspecific competition drove down total allocation to reproductive dispersal (Figure 2). This relationship is contrary to our hypothesis that competition will trigger an allocation to dispersal as an escape from adverse conditions (Colbert et al. 2009). There was no change to allocation to reproductive dispersal when *P. lacerum* was experiencing intraspecific competition with a genetic clone of itself (Figure 2). The difference in response to intraspecific and interspecific interaction suggests that *P. lacerum* is able to integrate information about its neighbors and respond accordingly (Boddy, 2000; Heilman‐Clausen & Boddy 2005). Competitor‐dependent differences in the expression of reproductive strategies are consistent with predictions from informed dispersal theory where an organism is able to assess its local environment (Clobert et al., 2009). However, we did not observe the increased allocation to reproductive dispersal as a response to competition that is expected under this theory.

### 4.1 Trade‐offs in allocation

We observed a trade‐off between growth and pycnidia production in *P. lacerum *(Figure 3), where increasing pycnidia production came at the cost of colony growth. Trade‐offs occur when it is not possible for evolution to optimize two functions simultaneously (Stearns, 1992). In this system, *P. lacerum* has an initial high allocation to growth rate prior to a switch to allocation to reproductive dispersal (Moving left on Figure 3). Gilchrist, Sulsky, and Pringle (2006) present an evolutionary optimality model of this behavior. In the Gilchrist model, increasing mycelial density as a result of fungal growth results in a decrease in local resources. When growth reaches a point where resources drop below a certain level, a switch to allocation to dispersal maximizes spore production for a given mycelial density (Gilchrist et al., 2006). The fact that we observed this in our study system suggests that *P. lacerum* may have a high initial allocation to growth to maximize the capture of territory, and thus maximize resource uptake before switching to a reproductive dispersal strategy to locate more resource patches (Heaton, Jones, & Fricker, 2016). However, when faced with competition, allocation purely to dispersal may leave a fungus with insufficient resources to defend itself against the attack from a neighboring fungus.

**Figure 4:**
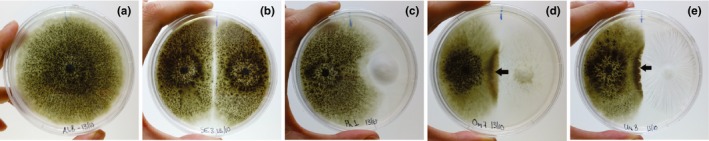
*Phacidium lacerum *interactions at 180 hr: (a) Alone, (b) Intraspecific competition, (c) *Phanerochaete* sp., (d) *Omphalotus *sp., (e) Isolate HWK5. Note the melanization of the hyphal front of *P. lacerum* (left) in (d), (e)

In our study, *P. lacerum* had similar slopes for the relationships for trade‐offs in allocation to dispersal and growth (Figure 3) when grown alone and when experiencing both intraspecific and interspecific competition apart from the *Phanerochaete *sp. treatment. However, *P. lacerum* responded to interaction with *Omphalotus *sp. and Isolate HWK5 with a reduced allocation to dispersal (Figure 2), suggesting that total allocation to dispersal shifts depending on the identity of the competitor. When *P. lacerum* was in competition with *Phanerochaete *sp., it had a higher slope for the trade‐off between pycnidia and growth (Figure 3) but had fewer total pycnidia (Figure 2), likely owing to the reduced size of the *Phanerochaete *sp. colony (Figure 4c). In line with the Gilchrist model, *P. lacerum* colonies could continue growing before occupying all available space and switching to dispersal to maximize the production of spores. On the other hand, when experiencing competition with *Omphalotus *sp. and Isolate HWK5, *P. lacerum* colonies responded with prominent pigmentation of the leading hyphal front in contact with the neighboring colony (Figure 4d,e).

Pigmentation of hyphae (predominantly with melanin) is a response commonly associated with defence against oxidative attack (Butler & Day, 1998; Butler, Gardiner, & Day, 2009; Hiscox, Baldrian, Rogers, & Boddy, 2010). The absence of this defensive response in the focal species when interacting with *Phanerochaete* sp. and a genetic clone of itself suggests that this defensive response is triggered only in the presence of certain competitors. The expression of defences in response to external stimuli is a phenomenon seen in plants known as induced defence (Agrawal 1999). The expression of these defences in the absence of attack is costly but prove to be beneficial for the persistence of an individual experiencing herbivory or parasitism (Agrawal 1999; Agrawal, 2001). In a fungal context, the expression of defence in a colony is in response to the presence of another fungal colony rather than direct mycophagy or parasitism. But the melanization of hyphae against fungal competition achieves a similar goal. Without the pressure of combative antagonism by a neighboring colony, a fungus is unlikely to melanize their hyphae as a defensive response. Allocation purely to reproductive dispersal in the face of competition will likely result in colony demise prior to successful dispersal if a colony has not adequately defended itself from attack by a neighbor. Conversely, expending resources to upregulate defence in the absence of a competitor will reduce resources available for allocation to reproduction (Agrawal 1999; Heaton et al., 2016). Thus, allocation to defence against attack by neighboring fungal colonies may trade‐off with allocation to dispersal or growth (Siletti, Zeiner, & Bhatnagar, 2017). Our research tends to support this alternative explanation of our observations, but further experimentation is needed to ascertain the true effects of competition on the observed trade‐off between growth and dispersal.

The exclusion of competitors by allocation to defence may allow a fungus to exhaust present resources confined within a territory and persist within the local patch, before switching strategies and dispersing to find new accessible resource patches. Allocation to growth or defence to the deficit of dispersal is not a viable strategy due to the dynamic nature of resource patches, and eventually most fungi must allocate to dispersal or risk local extinction (Thomas, 1994). Thus, the allocation in reproduction and dispersal in fungi will likely be context dependent and may be optimized when considered under a game theoretic framework (Kozlowski 1992), with shifts in allocation depending on the quality of a present patch. Due to the limitations of the present study, how allocation to defence affects the allocation to dispersal should be examined in greater detail in future studies.

## 5 CONCLUSION

Microbial metacommunities are receiving a large amount of recent attention (Fuhrman, 2009; Fukami et al., 2010; Hiscox, Savoury, Müller et al., 2015; Maynard et al., 2018; Nemergut et al., 2013; Peay, Bruns, Kennedy, Bergemann, & Garbelotto, 2007; Prosser et al., 2007; Van der Wal et al., 2016), and dispersal is increasingly identified as a key unknown with respect to the maintenance and continuity of these communities in a dynamic landscape (Calhim et al., 2018; Davison et al., 2018; Kneitel & Miller, 2003; Lancaster & Downes, 2017; Smith, Steidinger, Bruns, & Peay, 2018). We have demonstrated that fungi can acquire information from the environment, subsequently affecting changes to their allocation strategies to dispersal when faced with competition within the confines of the trade‐off between growth and dispersal. Understanding how decisions to disperse are weighed against (meta‐) community interactions will be a major step forward in predicting the development of these communities over time. The interactions within a community bound within an ephemeral resource island will shape the progression of species (Maynard et al., 2018), and dispersal will be the bridge that connects the stochastically formed islands in the environment. With allocation to dispersal being such a key component of life history theory, bridging the theory from life history theory (e.g., Gilchrist et al., 2006, Heaton et al., 2016) to microbial communities will help us begin to understand the complex patterns in community assembly we are observing.

## AUTHORS’ CONTRIBUTION

JC and WC conceived the ideas and designed methodology; WC, SB, and JP supervised the work of JC; JC collected data; JC and WC analyzed data; JP assisted with statistical analysis; JC lead the writing of the manuscript; All authors contributed critically to the drafts and gave final approval for publication.

## Data Availability

Data for this article can be found at the Dryad data repository (https://doi.org/10.5061/dryad.sh629f6).
