# Supplementary material for: When to cut your losses: Dispersal allocation in an asexual filamentous fungus in response to competition
Source: Ecol Evol. 2019 Mar 12;9(7):4129–37. doi: 10.1002/ece3.5041 (PMC6467841; doi:10.1002/ece3.5041)
Supplement: Supplementary file 1 [file ECE3-9-4129-s001.docx]

**SUPPLEMENTARY MATERIAL**


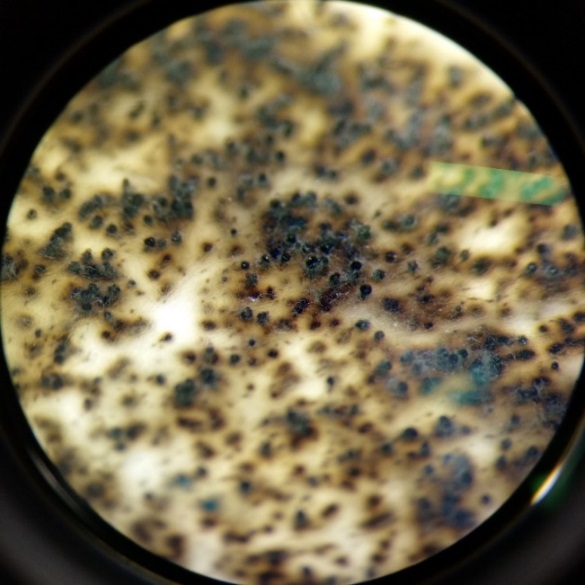

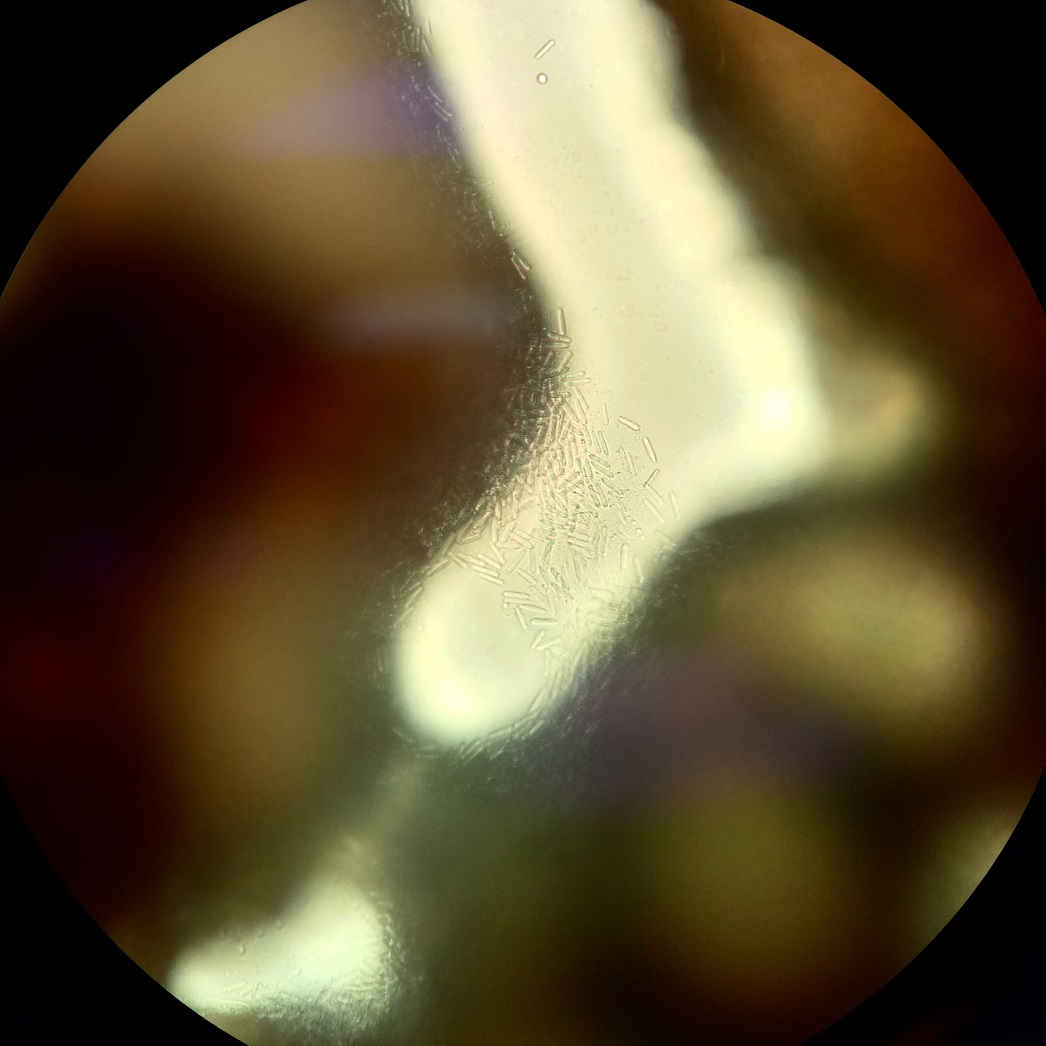


Supplementary Figure 1. **A)** *Phacidium lacerum* (Face008) pycnidial conidiomata, **B)** Spores extracted from pycnidia.
